# Assessing Patient Use of and Attitudes toward eHealth Services for Communication with Primary Care Centers in Saudi Arabia and Factors Affecting Usage

**DOI:** 10.3390/healthcare12191929

**Published:** 2024-09-26

**Authors:** Tourkiah Alessa, Khalid Alhussaini, Luc de Witte

**Affiliations:** 1 Department of Biomedical Technology, College of Applied Medical Science, King Saud University, Riyadh P.O. Box 10219, Saudi Arabia; 2 Center of Expertise Health Innovation, The Hague University of Applied Science, 2521 EN Den Haag, The Netherlands; l.p.dewitte@hhs.nl

**Keywords:** online communication, primary care, digital health, eHealth services, health technology adoption, Saudi Arabia healthcare

## Abstract

Background: This study investigates patients’ use of eHealth services, their awareness of the availability of these services, and their intention to use them in primary care. It also examines patient characteristics and factors that influence the use of these services. Methods: A cross-sectional design using questionnaires was conducted. Based on the unified theory of acceptance and use of technology (UTAUT), the participants rated the two most common services. Descriptive analyses and linear correlation analyses were performed. A simple linear regression was conducted to identify factors influencing the participants’ intention to use eHealth services. Results: In total, 1203 participants with an average age of 43.7 years were surveyed. The participants’ usage rates varied, with the lowest at 2.4%, for measuring vital signs, and the highest at 47.4%, for booking appointments. The intentions to use the services ranged from 22.5%, for video consultations, to 46.6%, for prescription refill requests. Approximately 20% of the respondents were unaware of each service’s availability. Positive associations were found between all the constructs and the intention to use online services, with a younger age being the most significant factor. Conclusions: The use of and intention to use eHealth services varied greatly. The participants were often unaware of the availability of these services. Promoting the availability and benefits of eHealth services could enhance patient engagement in primary care settings.

## 1. Introduction

The integration of internet and communication technologies into healthcare (eHealth) is becoming an increasingly important feature of healthcare systems around the world. These advancements have dramatically transformed healthcare delivery and access. Technologies such as mobile applications (apps), digital record systems, and telemedicine have garnered widespread recognition for their ability to improve access to healthcare, enhance patient outcomes, and lower costs [1,2]. These technologies have become particularly significant in primary care, streamlining the management of medical records and enhancing the accessibility of services. Enabling digital consultations, simplifying appointment bookings, and supporting the regular monitoring of patient health, eHealth has also become integral to care delivery and improving the experiences of patients and practitioners while expanding coverage, especially in areas with limited access to conventional healthcare infrastructure.

In response to these developments, Saudi Arabia has acknowledged the pivotal function eHealth might play in its healthcare reform initiatives, including those set out in the Vision 2030 plan. Through Vision 2030, Saudi Arabia plans to transform its economy and improve public services, including in the key domain of healthcare [3,4]. As part of these efforts, the Kingdom has placed a strong emphasis on eHealth, recognizing its potential to boost the quality of healthcare, ensure greater equality of access, and improve service efficiency [5].

Previous research has charted Saudi Arabia’s efforts to integrate eHealth technologies, especially in its primary care system [4,6,7]. Technologies that enable patients to engage digitally with healthcare providers are now available across most primary care centers (PCs) in Saudi Arabia.

The implementation of eHealth is expected to bring several benefits, including greater efficiency, quality of care, and patient satisfaction [8,9,10,11,12,13,14]. For example, online message systems reduce the need for face-to-face consultations and enhance communication between practitioners and patients, and the usefulness and convenience of these systems have received positive feedback from patients themselves [13,15,16]. Despite these advantages, research indicates that these systems are yet to be fully integrated into healthcare, and their adoption and use among patients remains inconsistent [17,18,19]. Alshammari reports that despite government efforts to promote their use, the adoption of eHealth across Saudi Arabia has been slower than expected [4]. Low awareness of these systems among patients is one factor that could be contributing to this. Whether patients intend to use eHealth has also been suggested as a factor impeding wider integration [4,9,20]. Developing a deeper understanding of patients’ intentions toward and the actual use of eHealth systems is crucial for improving their uptake.

In assessing patients’ willingness to adopt specific eHealth services, it is essential to consider a variety of factors, which might be physical, psychological, or social, alongside the specific needs of actual patients [17]. Gaining insights into these factors is also critical for the future effective adoption and usage of such technologies [17].

The Technology Acceptance Model (TAM) is widely regarded as one of the most reliable frameworks for evaluating the acceptance of emergent technologies [21]. In the TAM framework, two primary beliefs—perceived usefulness and perceived ease of use—serve as key predictors of a user’s intention to engage with a particular technology [21]. However, the process of adopting technology in healthcare settings is often more complex and is impacted by other contextual and social elements. To account for this complexity, the Unified Theory of Acceptance and Use of Technology (UTAUT) was developed, which extends the TAM framework to include further factors, including facilitating conditions and social influence, offering a greater level of detail to provide more rounded insights into this topic [22]. This extended model is valuable when seeking insight into the impact of environmental conditions, peer influence, and organizational support on patients’ readiness to embrace eHealth systems.

Previous research has also underscored the significant role that trust plays in users’ acceptance of new technologies in healthcare, a factor that is not adequately accounted for in either the TAM or the UTAUT. Or and Karsh’s (2009) review highlights the importance of trust as a predictor of technology acceptance in other areas and suggests this should also be considered in the context of healthcare [17]. In addition, patients’ intentions to engage with new technologies depend on other attitudinal factors in relation to technology, including their perceptions, predispositions, and emotions [23]. Consequently, this study integrates the UTAUT model along with the additional factors of trust and attitude to provide a fuller understanding of the factors influencing eHealth acceptance, building on previous research that has highlighted the significance of these as predictors [17,23]. This study retains a strong theoretical basis by combining these frameworks, providing a nuanced understanding of this topic while contributing to wider discussions on the various factors that drive or impede eHealth adoption.

The rapid advancement of digital technologies has transformed healthcare delivery globally, positioning eHealth as an essential element of contemporary healthcare systems [8,16]. In Saudi Arabia, substantial investments in healthcare infrastructure and the strategic focus on Vision 2030 offer significant potential for harnessing these innovations [4,5]. Nevertheless, despite these efforts, the adoption of eHealth services has not progressed as quickly as expected [6,7,24].

Moreover, while eHealth is gaining increasing prioritization and promotion globally, there has been limited research on users’ intentions to adopt these systems in primary care settings [9,16], with an even greater lack of research specific to Saudi Arabia and the other Gulf countries [4,6,24].

It is imperative, therefore, to fill this gap by studying this emerging topic area to investigate patients’ use of eHealth services, their awareness of the availability of these services, and their intention to use them in primary care, as well as examining characteristics and factors that influence their use. It is crucial to note that “intention to use” refers to a plan or willingness to use these services, while “usage” indicates the actual use of eHealth services [16]. This contributes to a comprehensive understanding of how technological services can be efficiently integrated into the nation’s healthcare system, providing crucial insights for policymakers and healthcare providers to enhance accessibility and efficiency in healthcare delivery [16,25,26].

The primary objective of the current study is therefore to investigate patients’ use of eHealth services, their awareness of the availability of these services, and their intention to use them in primary care. The secondary objective is to examine the patient characteristics and other factors that influence patients’ attitudes toward and use of two specific eHealth services: online appointment booking and asking questions online via the care center website or app. These two eHealth services were chosen for many reasons. First, they constitute the most important interactions in healthcare delivery for increasing patient engagement and improving health outcomes. The selected services play a central role in today’s healthcare context by addressing vital and routine aspects of patient care [16,25]. In particular, online appointment booking is crucial for streamlining and improving healthcare access, enhancing patient convenience, and reducing waiting times [3,24]. This service aligns with the increasing focus on digital solutions to boost efficiency and patient satisfaction in the Saudi healthcare system. Similarly, the opportunity to ask healthcare professionals questions online enables timely, direct communication between providers and patients, which is essential for supporting informed decision-making and addressing healthcare concerns [7,16]. As these services become more integrated into the country’s healthcare system, studying their adoption is particularly timely. With the field evolving toward supporting patient-centered care and digital transformation, understanding their impact and uptake is essential [6,24,27].

This study is original in examining Saudi healthcare using the TAM and UTAUT models and promises novel findings regarding factors that influence eHealth adoption and use within this specific cultural context. In the context of Saudi healthcare, where organizational support and cultural norms are considered to play an important role, both models are perfect for this research because they offer a thorough understanding of how external factors, along with considerations of attitudes and trust, affect patient adoption of eHealth services [16,24,28,29,30]. These models tackle the complex aspects of technology adoption that move beyond individual opinions [22,23].

The remainder of this paper is organized as follows: The subsequent section reviews the relevant literature on this topic area and outlines the hypotheses of the research. The next section outlines the methodology, providing detailed explanations of the study design, data collection methods, and data analysis techniques. This is followed by the results section, which presents the study’s key findings. Lastly, the conclusion highlights the limitations and implications of the study and provides the main recommendations for additional studies in this area.

## 2. Literature Review

The study of eHealth has gained significant importance in the past few years, partly due to the demands placed on healthcare systems to reach and treat expanding populations, like those in Saudi Arabia. Researchers and policymakers have highlighted eHealth systems as a key mechanism to improve healthcare efficiency and scale. While various studies have explored different dimensions of eHealth and its adoption, further research is required to obtain more views about the integration and impact of these technologies [4,30,31].

One systematic review examined the uptake of eHealth systems globally over the last five years, showing that nations with strong governmental backing and well-defined regulatory frameworks tend to have more success in rolling out eHealth services [32]. This review underscored the essential role of involving patients in the design and rollout process and the need to include user feedback to ensure that these services meet their needs and are user-friendly. Research studies conducted in the Netherlands found a disparity between patients’ lack of actual usage of eHealth to communicate with healthcare providers and a high intention to use such systems. A lack of awareness about the availability of these systems at primary care centers was identified as a significant barrier to use, suggesting better uptake could be achieved through improved awareness campaigns [32].

Studies in MENA countries, such as the UAE, Egypt, and Turkey, have examined the obstacles to eHealth implementation in developing countries, identifying key regulatory constraints, cultural barriers, trust deficits, and user attitudes as key issues that must be tackled to improve the uptake of eHealth services in these study contexts [33]. This highlights the importance of promoting awareness and addressing cultural barriers to foster the adoption of eHealth services [33].

Moreover, studies examining the Saudi context, which consider citizens’ perceptions, preferences, and experiences with eHealth services, including telemedicine, [24,29,30,31] have shown that a lack of awareness continues to impede patients’ acceptance and utilization of these technologies [31]. Several other key factors have shaped individuals’ intentions to use eHealth systems, including perceptions of their usefulness, perceived cost barriers, and privacy concerns [26,29,30,34,35]. These researchers also identified perceived ease of use as a further factor indirectly influencing patients’ attitudes.

Although the successful implementation of eHealth technologies depends on properly understanding patients’ openness, research has pointed out that relatively little investigation into the implementation and uptake of these new technologies in Saudi Arabia considers the perspectives of its various stakeholders, including patients’ own perspectives [7,36]. This highlights the urgent need for further research to explore patients’ awareness and perceptions of and intentions to use eHealth systems. Additionally, only a few studies [24,29,30] have examined the factors affecting the adoption of eHealth among the Saudi public based on theories such as TAM and the Theory of Planned Behavior (TPB). These studies determined that patients’ own subjective attitudes and cultural norms were among the predictors of their intentions to use such systems. However, these studies focused only on patients outside the Saudi primary care system, leaving a gap in research examining eHealth implementation within primary care [24,29,30]. This topic requires further study, utilizing models such as the UTAUT to understand the factors influencing Saudi primary care patients’ intentions to engage with eHealth technologies.

### 2.1. Research Model and Hypotheses Development

UTAUT is regularly used to understand and predict the acceptance and use of new technological innovations [22,28]. This study uses UTAUT alongside the additional factors of trust and attitude to provide greater insight into the factors that affect patients’ acceptance of eHealth technologies within the Saudi context.

The UTAUT framework recognizes four primary factors that influence an individual’s intention to adopt a new technology:

1. Performance Expectancy (PE): The belief that the technology will enhance performance.
2. Effort Expectancy (EE): The perceived ease associated with using the technology.
3. Social Influence (SI): The belief that influential others think the technology should be used.
4. Facilitating Conditions (FC): The perception that adequate support and resources are available to facilitate one’s use of the technology.

The above factors are hypothesized to directly impact individuals’ intention to use eHealth services, in turn impacting individuals’ actual usage. Additionally, the model for the current study includes the supplementary factors of trust and attitude to offer fuller insights into the user characteristics that might impede or promote engagement with eHealth systems.

### 2.2. Hypotheses

Drawing on the UTAUT framework and supported by a review of relevant literature, the current study poses the following hypothesis:

Performance Expectancy (PE), Effort Expectancy (EE), Social Influence (SI), Facilitating Conditions (FC), Trust, and Attitudes have a positive influence on patients’ intentions to engage with eHealth services, such as booking appointments through a mobile application/website or asking healthcare professionals questions via an app/website.

Many key factors have been identified as key drivers for the adoption of eHealth services, each significantly influencing patients’ willingness to engage with these technologies. First, users are more likely to adopt eHealth services if they perceive that these technologies will improve their healthcare experience, with perceived usefulness significantly influencing the intention to use eHealth services [24,33]. Ease of use also constitutes a crucial role, particularly for older people and those with lower digital literacy, which affects adoption rates in rural areas [29,30,33,36]. Additionally, positive attitudes and social influence strongly impact adoption [27,28,29,30]. Facilitating conditions, such as reliable internet access and user-friendly platforms, are essential for adoption, particularly in regions with limited infrastructure [31,37]. Lastly, trust in digital healthcare providers plays an important part, especially in areas where privacy concerns are prevalent [17,31].

Although eHealth technologies are increasingly being adopted and prioritized within modern healthcare systems globally, there remains a significant lack of research examining patients’ attitudes toward eHealth technologies and their actual use of eHealth systems in Saudi primary care contexts and across the Gulf countries in general. This study aims to fill this gap in our knowledge of eHealth integration by using an adapted UTAUT framework to investigate the factors impacting usage intentions and engagement with eHealth in Saudi primary care settings.

## 3. Methods

### 3.1. Design and Participants

This research utilized a cross-sectional study design. Questionnaires were given to participants in the main/biggest PC in Riyadh, the capital city of Saudi Arabia, and its largest population center. The PCs are open 24 h a day, seven days a week, and treat patients from across the city.

Participants were recruited from February 2022–August 2022, using systematic random sampling, whereby the study researcher spoke with every third person who entered the waiting area at the PC to assess their interest in participating and suitability. The inclusion criteria were adults, aged 18 years and over, who had contacted their PCs at least once in the year before sampling. Prior to their participation, participants’ consent was obtained. Ethical approval for conducting this study was obtained under approval number 21-518E from the Ministry of Health (MOH) in Riyadh, Saudi Arabia.

According to statistics from the Saudi MOH, the number of Saudi visitors to PCs in Riyadh City was 7,372,405. Given the size of this population, the sample size was set at 384 participants, given a confidence interval of 95%, a 0.05 alpha error, ±5% accuracy, and power of 80%. This sample size was calculated using Cochran’s formula [38]. The target sample size for this study therefore ranges from 600 to 1200 eligible participants to ensure greater precision and reliability in the findings.

### 3.2. Measurements

#### 3.2.1. Questionnaire

The questionnaire used to establish respondents’ actual use of eHealth and their intention to use such services is derived from previous research, where it has been utilized successfully [9,16]. It consists of three main sections: participants’ characteristics; use, intention to use, and the availability of the eHealth services; and factors affecting their intention to use (See Supplementary Materials).

The questionnaire was translated into Arabic and validated according to the 258 WHO Process of Translation and Adaptation of Instruments. The researcher translated 259 into Arabic, and a professional service back-translated it into English to ensure accuracy. 260 A pilot study with patients confirmed the reliability of the translated questionnaire, 261 showing a Cronbach’s alpha of 0.9 and a scale-level content validity index (S-CVI) of 0.95. 262 These results indicate that the questionnaire is both reliable and valid for use in the target 263 population.

#### 3.2.2. Participants’ Characteristics

Data on participant characteristics were gathered, including their gender, age, education level, geographical location within Riyadh, their internet use, and whether they rated the internet as difficult or easy to use.

#### 3.2.3. Use and Intention to Use and Availability of Internet Services

The questionnaire collected data on participants’ actual use of eHealth, their awareness of the existence and availability of such services, and their intention to use such services when communicating with their healthcare providers about the following:

(1) appointment booking using website/app; (2) receiving SMS appointment reminders; (3) asking questions of healthcare professionals using website/app; (4) taking home measurements of vital signs including blood pressure and weight, and sending these to a healthcare professional via website/app; (5) requesting repeat prescriptions via website/app; (6) accessing their medical data online; (7) video consultation via the app/web.

#### 3.2.4. Factors Influencing Intention to Use eHealth Services

To understand which factors influence the participants’ intention to use two eHealth services: (1) appointment booking using a website/app and (2) asking questions of healthcare professionals using a website/app. Participants were asked to rate several items divided according to six subscales based on the UTAUT model as well as recommendations from studies by Or and Karsh (2009) and Spil and Schuring (2006) [17,23]. These subscales were effort expectancy, trust, attitude, facilitating conditions, social influence, and performance expectancy.

### 3.3. Data Analysis

Descriptive analyses were used to calculate participant characteristics and to examine participants’ actual use, intention to use, and awareness of availability regarding the seven eHealth services.

Spearman Correlation Tests were conducted to assess the correlation between the six subscales (effort expectancy, attitude, trust, performance expectancy, social influence, and facilitating conditions). Strong correlations between the six subscales were found, indicating significant multicollinearity, which violated the assumptions required for multiple regression analysis. The variance inflation factor (VIF) values were examined, confirming multicollinearity. Hair et al. (2010) found that VIF values of 5 to 10 imply significant multicollinearity [39]. Due to the presence of significant multicollinearity, conducting multiple regression analysis was not feasible. Hence, in order to examine how each individual characteristic (such as age, gender, and education level) and subscale (such as attitudes, perceived usefulness, perceived ease of use) related to participants’ intention to use two specific eHealth services, a series of simple linear regression analyses were conducted. Each analysis focused on a single dependent (intention to use one of the two eHealth services) and a single independent variable (e.g., a construct or individual characteristic such as age).

## 4. Results

Out of 1650 participants approached, 1250 responded to the questionnaire. Among these 1250 respondents, 47 participants were excluded because they did not complete all of the questionnaire items regarding their use, intentions, and awareness of internet services. Therefore, the total number of participants included is 1203. Table 1 shows participant characteristics for the study (n = 1203).

### 4.1. Use, Intention to Use, and Awareness of Availability of Internet Services

**Figure 1:**
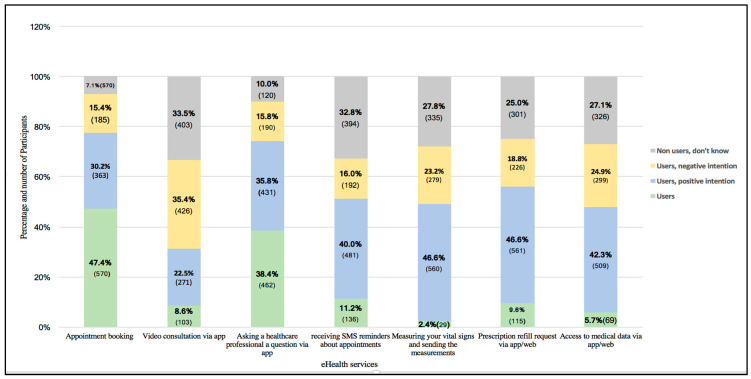
Number of participants that use eHealth services and their intention to use eHealth services.

As shown in Figure 1, the use of most of the eHealth services (5 out of 7) was low. Very few of the participants reported having measured their vital signs and sent the measurement to their healthcare professional (2.41%, 29/1203) or having accessed their medical data via the app/web (5.74%, 69/1203). A larger number of participants had undertaken a video consultation via the app in the past year (8.56%, 103/1203), requested a prescription refill via the app (9.56%, 115/1203), or received SMS reminders about appointments (11.31%, 136/1203). The other two services were used more frequently. Booking an appointment via the app/web was the most frequently used (47.4%, 570/1203), followed by asking a healthcare professional a question via the app (38.4%, 462/1203).

Participants were also asked about their intentions to use eHealth services in the future, and these results are also presented in Figure 1. The highest percentages of participants with a positive intention were found for prescription refill requests (46.63%, 561/1203), measuring their vital signs and sending the measurement (46.55%, 560/1203), followed by having access to medical data (42.31%, 509/1203) and receiving SMS appointment reminders (39.98%, 481/1203). Approximately a third of participants (35.5%) and (30.2%) reported positive intentions regarding their future use of asking healthcare professionals and appointment booking, respectively.

The proportion of respondents expressing a negative intention toward future eHealth use ranged from (15.4%, 185/1203) for online appointment booking to (35.41%, 426/1203) for video consultations. For most services, more than a quarter of respondents said that they did not know whether they would like to use internet services in the future, ranging from (25.0%, 301/1203) for prescription refill requests to (33.50%, 403/1203) for video consultations.

**Figure 2:**
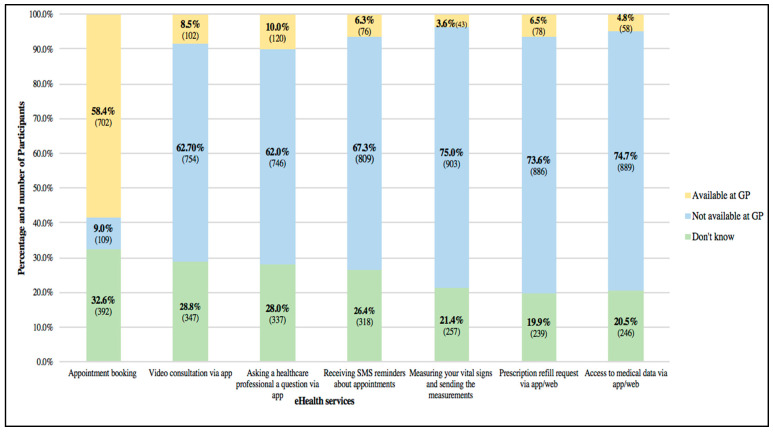
Participants’ awareness of the availability of eHealth Services.

Figure 2 shows participants’ levels of awareness of the availability of each of the internet services at their primary care center. Measurement and transmission of vital signs had the lowest level of awareness (3.57%, 43/1203) whereas online appointment booking had the highest (58.3%, 702/1203). When asked which services they thought were unavailable at their PC, (9.06%, 109/1203) reported that online appointments were not possible, and (75.06%, 903/1203) reported that it was not possible to measure and share vital signs. Many patients did not know whether any of these online services were available at their PC (around one-fifth of respondents for each service).

### 4.2. Relationship between Factors and Intention to Use eHealth Services

Table 2 and Table 3 present the correlation between the constructs (effort expectancy, attitude, trust, performance expectancy, social influence, and facilitating conditions) and the two selected eHealth services. Correlations between both services and each of the studied constructs were statistically significant, higher than or equal to r = 0.64 (*p* < 0.001). For the constructs that could influence the intention to ask questions via the app or website, most coefficients were 0.80 or above, as shown in Table 2. In order to assess the interrelatedness of the constructs, variance inflation factors (VIFs) were calculated. All these VIF values were between 5 and 10, indicating strong multicollinearity.

For the constructs that influence participants’ appointment booking, 4 correlation coefficients exceeded the value of 0.80: attitude and effort expectancy (r = 0.85), performance expectancy and attitude (r = 0.84), each with a VIF value between 5 and 10, indicating high multicollinearity. Consequently, the strong multicollinearity makes multiple regression analysis impossible.

Table 4 presents the simple linear regression analysis results. Each of the studied constructs (effort expectancy, attitude, trust, performance expectancy, social influence, and facilitating conditions) was significantly associated with the intention to book appointments via the app and to ask healthcare professionals questions via an app/website. For asking healthcare professionals online questions, the R ranged from 0.34 for trust and facilitate condition to 0.45 for performance expectancy. The R for booking appointments via the app/web ranged from 0.24 for trust to 0.36 for attitude.

The characteristics of participants, age, and internet usage showed a significant correlation with the intention to book appointments online and ask healthcare professionals questions via the app or website. The Rs for age were 0.48 and 0.56, respectively, and the Rs for internet usage were 0.28 and 0.21, respectively. In Table 4, the analysis reveals that all six subscales have significant correlations with the intention to use the services but also that age is the main explaining variable.

## 5. Discussion

Participants’ use of and intention to use eHealth services to contact healthcare professionals varied greatly between each of the services studied. Online appointment booking was used most frequently (47.4%) while measuring vital signs and sending the measurements to healthcare professionals was the least frequent (2.4%). Participants who had not used these services at least once in the past year expressed differing positive intentions toward using them in the future, ranging from 46.6% for prescription refill requests via the app/website and measuring vital signs and sending them to healthcare professionals to 22.5% for video consultation. Many participants (around a fifth for each service) did not know whether these services were available at their PC. The study also investigated the participant characteristics and other factors that could influence participants’ intention to use eHealth services to communicate with their PC. Several of the studied constructs exerted a significant influence on participants’ intention to use online appointment booking, namely effort expectancy, performance expectancy, trust, attitude, facilitating conditions, social influence, the characteristics of age, and internet usage. However, age is the main explanatory variable. Given the relatively small number of participants who reported not knowing their intentions, combined with the relatively high correlations between the constructs measured, the findings suggest that study respondents had clear and consistent views regarding the use of eHealth services.

Although the findings of previous research [16,18] indicate that the use of eHealth services is low, the present study found them to differ from one service to another. One reason for this variance may be that Saudi people were urged to use these services during the COVID-19 lockdown [25,26], resulting in greater familiarity with these services and ease of use [3]. Moreover, the Ministry of Health (MOH) has also encouraged patients to book appointments via an app to reduce their waiting time and to serve them quickly, and to ask a doctor a question via the app/website when no appointments are available) [3,27]. These drives could explain why these two services are used more than the others.

Our study confirms the findings of previous research, which has concluded that often patients do not know about the existence of eHealth applications or they are not aware of the possibilities of these applications [4,34]. Participants’ lack of knowledge about service availability will inevitably influence their usage. In the current study, only 10% of respondents thought that it was possible to ask questions of healthcare professionals online, and many replied that they did not know or that eHealth services were not available when asked about the availability of these eHealth services, despite these being available at a large proportion of primary care centers [5,25,31]. Patients’ awareness and use of eHealth services has greatly increased following the COVID-19 pandemic [35,40] with one study reporting that the number of online medical consultations in China was 20 times greater in 2020 than in the previous year [41]. However, many of the eHealth solutions implemented in Saudi Arabia during this time were temporary, and have been replaced with new ones that patients may not know. Mair et al. (2015) highlighted in their review the importance of communicating the purposes, benefits, and values of eHealth services to prospective users as a way to improve the implementation of eHealth solutions [28], further indicating that lack of awareness was likely to have influenced respondents’ use and intentions in the present study [32,42].

Although all of the studied constructs were found to exert influence over participants’ use of and intention to use eHealth services, most of the independent constructs exhibited high correlations. A previous study (in the Dutch context) applied the UTAUT model to examine participants’ intention to use eHealth services [16], finding moderate to high correlations between constructs similar to the present study. On the other hand, a few studies have utilized a modified version of the UTAUT model to examine patients’ attitudes toward internet services for patient self-management and did not report high correlations between independent constructs [17,43,44]. A current research study indicated that culture plays a key role in the strength of relationships among the model constructs [28].

Patient characteristics have been shown to influence their intention to use eHealth services, and this relationship has been well studied [45,46]. The results of the present study show that older participants and those who reported greater difficulty with internet use in general also expressed more negative intentions regarding eHealth. Most previous studies have reported the same patterns [47,48]. Whereas it has been suggested by some researchers that the association between age and technology usage will become less pronounced as the population develops greater familiarity with internet communication technologies [43], a study by Heart and Kalderon (2011) found that greater adoption of ICT among older participants did not necessarily translate into greater adoption of eHealth services [49]. Among their study population, the most commonly reported reason not to use eHealth services was that they were perceived to be unnecessary, underscoring the importance of communicating the potential benefits of these technologies [16].

### Strengths and Limitations

A strength of this study is that it is the first to investigate participants’ use of and intention to use eHealth services in the context of Saudi Arabia and the wider Gulf Region, thus providing essential evidence regarding participants’ engagement with these technologies in PC settings. Second, a high number of participants (n = 1203) between 18 and 74 years of age participated. Due to the study’s concentration on an urban population, which might not accurately represent the experiences of people in rural areas, the results may only have limited generalizability. The study, however, provides valuable insights into other cultures and contexts similar to Saudi Arabia and the Saudi Ministry of Health.

However, there are some limitations to consider. The findings may have limited generalizability due to the study’s focus on an urban population, which may not fully represent the experiences and intentions of individuals in rural areas or those with different socioeconomic backgrounds.

The questionnaire used in this study was adopted from a previous study, where it was designed to gather data on participants’ intention to make appointments and ask questions online based on the validated UTAUT model. The trust and valuation subscales were not validated, as the intention was not to produce a new validated model predicting patients’ intentions, nor are the factors included claimed to be the only ones informing participants’ intentions to use these services. Instead, the study investigated possible factors influencing the general population’s intentions by using predictors suggested in previous research. Given the minimal representation of “do not know” responses, these were treated as missing data in the analysis to prevent potential skewing of the results.

## 6. Conclusions

The present study found that the use of and intention to use eHealth services varied greatly among the study population, depending on the specific service examined. Participants’ awareness of the availability of services was generally limited, which would inevitably influence both their use and intentions. While a substantial proportion of respondents expressed positive intentions about future use, many participants reported an unwillingness to use and engage with eHealth services. The practical implications of this study underscore the necessity of increasing public awareness and disseminating additional information to improve patients’ experiences with eHealth services. It is recommended that healthcare providers and policymakers prioritize patient education through awareness campaigns and educational initiatives and enhance the usability of these services through user input and feedback to make them more user-friendly. Future research should investigate patients’ long-term experience with eHealth services through longitudinal studies, monitoring changes in satisfaction, awareness, and utilization over time. It is crucial to take into account that different demographics from different regions might produce conclusions that are more widely applicable.

## Figures and Tables

**Table 1:** Participants Characteristics (n = 1203).

	Mean (SD) or n (%)
Age in years	43.7 (17.1)
Gender	
Male	535 (44.5%)
Female	668 (55.5%)
Level of education	
Low	479 (39.8%)
Medium	698 (58.0%)
High	26 (2.2%)
Internet usage (years)	
<1	201 (16.7%)
1–3	614 (51.0%)
>3	388 (32.3%)

**Table 2:** Matrix of linear correlations and variance inflation factor values between the independent constructs that could influence the intention to ask healthcare professionals a question via app/website.

Asking Healthcare Professionals, a Question via App/Website	EE	PE	TR	AT	FC	VIF Value
Effort Expectancy (EE)	1					7.3
Performance Expectancy (PE)	0.84	1				8.1
Trust (TR)	0.82	0.81	1			6.1
Attitude (AT)	0.85	0.84	0.82	1		8.1
Facilitating Condition (FC)	0.82	0.87	082	0.82	1	7.6
Social Influence (SI)	0.84	0.85	0.81	0.87	0.84	8.2

All results are found to be significant at the *p* < 0.01 level.

**Table 3:** Matrix of linear correlations and variance inflation factor values between the independent constructs that could influence intention to book appointments using website/app.

Appointment Booking Using Website/App	EE	PE	TR	AT	FC	VIF Value
Effort Expectancy (EE)	1					5.7
Performance Expectancy (PE)	0.75	1				5.5
Trust (TR)	0.70	0.72	1			4.3
Attitude (AT)	0.85	0.84	0.70	1		5.1
Facilitating Condition (FC)	0.70	0.79	074	0.78	1	5.7
Social Influence (SI)	0.72	0.76	0.64	0.78	0.74	5.1

All results are found to be significant at the *p* < 0.01 level.

**Table 4:** Simple linear regression of constructs and characteristics with intention toward using booking appointment planning and asking questions via app/website ^a^.

		Booking Appointment Planning				Asking Questions via App/Website		
Independent Variable	Participants Number	R	Unstandardized B	*p*	Participants Number	R	Unstandardized B	*p*
Effort Expectancy	1191	0.30	0.20	0.001	1195	0.42	0.24	0.001
Performance Expectancy	1187	0.32	0.20	0.001	1190	0.45	0.25	0.00
Trust	1994	0.24	0.15	0.014	1188	0.34	0.19	0.001
Attitude	1183	0.36	0.23	0.001	1192	0.40	0.23	0.001
Facilitating condition	1199	0.26	0.17	0.007	1200	0.34	0.19	0.001
Social Influence	1201	0.31	0.20	0.001	1197	0.42	0.23	0.001
Gender	1203	0.13	−0.11	0.18	1203	0.17	−0.13	0.08
Age	1203	0.48	−0.01	0.001	1203	0.56	−0.02	0.001
Level of education	1203	0.16	−0.12	0.105	1203	0.15	−0.096	0.13
Internet usage	1203	0.28	0.18	0.003	1203	0.37	0.21	0.001

^a^ All constructs and characteristics had a significant association with an intention to use both services, except for level of education and gender.

## Data Availability

The data presented in this study are not publicly available due to ethical restrictions. However, they are available from the corresponding author upon reasonable request.

## Supplementary Materials

The following supporting information can be downloaded at: https://www.mdpi.com/article/10.3390/healthcare12191929/s1, questionnaires.
