# Supplementary material for: Assessing Patient Use of and Attitudes toward eHealth Services for Communication with Primary Care Centers in Saudi Arabia and Factors Affecting Usage
Source: Healthcare (Basel). 2024 Sep 26;12(19):1929. doi: 10.3390/healthcare12191929 (PMC11475504; doi:10.3390/healthcare12191929)
Supplement: Supplementary file 1 [file healthcare-12-01929-s001.zip › healthcare-3166315-supplementary.pdf]

1. **What is your gender?**

  - o Male
  - o Female

2. **What is your age in years?**

  ………………….. years old

3. **What is your highest level of education?**

  - o High School or less
  - o Some Post-Secondary Education or Bachelor's Degree
  - o Master's Degree or Ph.D or equivalent

4. **How much is your experience with using internet?**

  - o <=1 years
  - o 1-3 years
  - o >3 years

A) <u>Actual use</u>

1. Have used the following eHealth services at the primary care centers.

**eHealth services**	**Yes**	**No**	**I do not know about this service**
Making/booking an appointment via app/web	❑	❑	❑
Asking a healthcare professional a question via app/web	❑	❑	❑
Reminder bout appointment via SMS	❑	❑	❑
Measuring your vital signs e.g., weight, blood pressure or blood sugar level at home and sending the measurement to your health-care professional via app/web	❑	❑	❑
Prescription refill request via app/web	❑	❑	❑
Video consultation via app/ web	❑	❑	❑
Access to medical data via app/web	❑	❑	❑

B) **Awareness of the availability of eHealth services at the primary care centers**

1. Whether you know if the following eHealth services are available at your primary care canter's or not?

	Available at Pcs	Not available at Pcs	I do not know
a. Making/booking an appointment	❑	❑	❑
b. Asking a healthcare professional a question via app/web	❑	❑	❑
c. Reminder bout appointment	❑	❑	❑
d. Measuring your vital signs e.g., weight, blood pressure or blood sugar level at home and sending the measurement to your health-care professional	❑	❑	❑
e. Prescription refill request	❑	❑	❑
f. Video consultation	❑	❑	❑
g. Access to medical data	❑	❑	❑

C) Intention to use

1- Do you think you would use one of the above-mentioned Internet applications in the future if you were offered the opportunity?

	Yes, definitely	Yes, probably	I don't know yet	No, probably not	No, definitely not
a. Making/booking an appointment	❑	❑	❑	❑	❑
b. Asking a healthcare professional a question via app/web	❑	❑	❑	❑	❑
c. Reminder bout appointment	❑	❑	❑	❑	❑
d. Measuring your vital signs e.g., weight, blood pressure or blood sugar level at home and sending the	❑	❑	❑	❑	❑

measurement to your health-care professional					
e. Prescription refill request	❑	❑	❑	❑	❑
f. Video consultation	❑	❑	❑	❑	❑
g. Access to medical data	❑	❑	❑	❑	❑

2. What do you expect from the following eHealth service if you need additional care in the future? To what extent do you agree with the following statements?

**Booking/Making an online appointment**	***Strongly disagree***	***Disagree***	***Agree***	***Strongly agree***	***I don't know***
a. Is easy to do	❑	❑	❑	❑	❑
b. Is easy to learn	❑	❑	❑	❑	❑
c. Is reliable	❑	❑	❑	❑	❑
d. Works well	❑	❑	❑	❑	❑
e. Is a pleasant way to interact with health-care professionals	❑	❑	❑	❑	❑
f. Fits easily into my daily routine	❑	❑	❑	❑	❑
g. Is something my family or friends would like to do	❑	❑	❑	❑	❑
h. Makes it easier to contact a health-care professional when I want	❑	❑	❑	❑	❑

3. What do you expect from the following eHealth service if you need additional care in the future? To what extent do you agree with the following statements?

**Asking a healthcare professional a question via app/web**	***Strongly disagree***	***Disagree***	***Agree***	***Strongly agree***	***I don't know***
a. Is easy to do	❑	❑	❑	❑	❑
b. Is easy to learn	❑	❑	❑	❑	❑
c. Is reliable	❑	❑	❑	❑	❑
d. Works well	❑	❑	❑	❑	❑
e. Is a pleasant way to interact with health-care professionals	❑	❑	❑	❑	❑
f. Fits easily into my daily routine	❑	❑	❑	❑	❑

**Asking a healthcare professional a question via app/web**	***Strongly disagree***	***Disagree***	***Agree***	***Strongly agree***	***I don't know***
g. Is something my family or friends would like to do	❑	❑	❑	❑	❑
h. Makes it easier to contact a health-care professional when I want	❑	❑	❑	❑	❑
